# Heat Transfer Performance of a 3D-Printed Aluminum Flat-Plate Oscillating Heat Pipe Finned Radiator

**DOI:** 10.3390/nano14010060

**Published:** 2023-12-25

**Authors:** Xiu Xiao, Ying He, Qunyi Wang, Yaoguang Yang, Chao Chang, Yulong Ji

**Affiliations:** Institute of Marine Engineering and Thermal Science, Marine Engineering College, Dalian Maritime University, Dalian 116026, China; xiaoxiu@dlmu.edu.cn (X.X.); ying12200@163.com (Y.H.); 1120211168@dlmu.edu.cn (Q.W.); yangyaoguang2023@163.com (Y.Y.)

**Keywords:** 3D-printed, flat-plate oscillating heat pipe, aluminum finned radiator, thermal resistance, heat transfer performance

## Abstract

As electronic components progressively downsize and their power intensifies, thermal management has emerged as a paramount challenge. This study presents a novel, high-efficiency finned heat exchanger, termed Flat-Plate Oscillating Heat Pipe Finned Radiator (FOHPFR), which employs arrayed flat-plate oscillating heat pipes (OHP) as heat dissipation fins. Three-dimensional (3D)-printed techniques allow the internal microchannels of the FOHPFR to become rougher, providing excellent surface wettability and capillary forces, which in turn significantly improves the device’s ability to dissipate heat. In this study, the 3D-printed FOHPFR is compared with traditional solid finned radiators made of identical materials and designs. The impacts of filling ratio, inclination angle, and cold-end conditions on the heat transfer performance of the 3D-printed FOHPFR are investigated. It is demonstrated by the results that compared to solid finned radiators, the FOHPFR exhibits superior transient heat absorption and steady-state heat transfer capabilities. When the heating power is set at 140 W, a decrease in thermal resistance from 0.32 °C/W in the solid type to 0.11 °C/W is observed in the FOHPFR, marking a reduction of 65.6%. Similarly, a drop in the average temperature of the heat source from 160 °C in the solid version to 125 °C, a decrease of 21.8%, is noted. An optimal filling ratio of 50% was identified for the vertical 3D-printed FOHPFR, with the minimal thermal resistance achieving 0.11 °C/W. Moreover, the thermal resistance of the 3D-printed FOHPFR is effectively reduced compared to that of the solid finned radiator at all inclination angles. This indicates that the FOHPFR possessed notable adaptability to various working angles.

## 1. Introduction

With the constant advancement of electronic components toward miniaturization and high power, thermal management has become a research hotspot currently [1,2,3]. As a key element of thermal management, heat dissipation technology has attracted a great deal of attention [4]. According to previous research [5], the failure rate of electronic devices increases exponentially with temperature. When the temperature is too high, there is a sharp decline in the reliability of electronic devices. Traditional heat dissipation solutions, including solid material thermal conductivity, natural convection, air-cooled, and water-cooled technologies [6,7], are difficult to meet the needs of high-power electronic products. OHP, as a promising cooling solution, is receiving increasing attention from researchers [8,9].

Akachi [10] was the first to propose the OHP technique. Based on their structural designs, OHPs are generally categorized into two types: the tubular OHP, which is a closed capillary bent into a serpentine structure, and the flat-plate OHP, featuring closed curved channels processed on a flat plate. Compared with tubular OHP, flat-plate OHP addresses the limitations of metal tubes, reduces channel spacing, and enhances structural compactness effectively. Thus, planar contact with electronic components can be achieved. Notably, it performs better in heat transfer and has a lower startup temperature, which makes it one of the optimal solutions to the thermal problems of electronic components [11,12]. In the past few decades, there have been many studies conducted on flat-plate OHP. According to the relevant research, the heat transfer performance of flat-plate OHP is affected by many factors. Currently, the research on this subject mainly focuses on the operational performance of flat-plate OHP given different working fluids [13,14,15], filling ratios [16,17,18], and inclination angles [19,20].

Via visualization and the heat transfer experiments conducted on flat-plate OHPs, Khandekar et al. [21,22] found that the heat transfer performance of horizontally placed flat-plate OHPs is poor and even worse under anti-gravity conditions. Jang et al. [23] developed flat-plate OHP by using hot graphite sheets. When the experimental inclination angle varied from 0° to 90°, the thermal resistance of flat-plate OHP decreased by 56% and 62%, respectively, compared to hot graphite sheets. However, its heat dissipation performance is still poor, and the applicable heating power is relatively low. Mehta et al. [15] investigated the performance of flat-plate OHP by using five different working fluids: deionized water, acetone, methanol, FC-72, and ethanol. As suggested by their research results, acetone has the lowest thermal resistance as a working fluid. However, the minimum thermal resistance of acetone as a working fluid is 0.39 °C/W, which makes it difficult to achieve an ideal heat transfer performance. In addition, improving the micro–nano structure of the channel can provide a strong capillary effect for the working fluid, which can significantly enhance the heat transfer performance of the flat-plate OHP [24,25,26,27]. Ji et al. [25] modified the copper surface with copper oxide particles, which led to a change in the wettability of the copper surface from hydrophilicity to superhydrophilicity. Compared with the original version, this modification reduces the thermal resistance of OHP by about 50%. Nevertheless, certain limitations persist, notably the limited area available for heat dissipation. Qu et al. [27] sintered a layer of porous copper powder suction core on the bottom of the copper-based flat-plate OHP channel. According to the experimental results, the porous structure is effective in reducing the starting temperature of the heat pipe and shortening the starting time. However, deterioration occurs in the performance of the flat-plate OHP sintered with copper powder suction core at high power, which is attributed to the limitations of large evaporation capacity and the filling capacity of the suction core. However, the heat transfer performance of flat-plate OHP with microstructure inner surface remains poor at high power. In addition, the traditional manufacturing process of flat-plate OHP is relatively complex and limited by processing technology, making it difficult to manufacture complex internal channels [24].

With the rapid development of 3D-printed technology in recent years, it has been increasingly popularized in the manufacturing industry [28]. By stacking the raw materials layer by layer, 3D-printed technology can be applied to accurately construct complex internal structures and shapes at once, opening up new possibilities for the improvement in heat pipe performance. Arai et al. [29] adopted 3D-printed technology to produce flat-plate OHP with transparent polyurethane as raw material, while Chang et al. [30] applied SLM (selective laser melting) technology to develop aluminum flat-plate OHP. The results indicate that these 3D-printed devices perform better in heat transfer, and 3D printing provides an effective solution to the integrated manufacturing of flat-plate OHP. In spite of this, the drawbacks of plate OHP are still not well addressed. Due to the insufficient heat dissipation area of a single flat-plate OHP and the inability to solve the high heat flux density of electronic components, we are considering whether 3D-printed technology can be incorporated to increase the number of flat-plate OHPs and improve heat dissipation performance. At present, there is relatively little research focusing on array plate OHP.

In order to further improve the performance of flat-plate OHP and meet the requirements of high-power equipment on cooling, it is proposed in this study to develop an advanced finned heat sink based on array flat-plate OHPs. Three-dimensional SLM technology is applied to achieve the lightweight and efficient production of aluminum-based FOHPFR. The internal microchannels of FOHPFR and its roughly sintered inner surface provide excellent surface wettability and a sufficient capillary force for heat pipe integration, which significantly improves the heat dissipation performance of electronic devices. In addition, this study explores the effects of various physical parameters on the heat transfer performance of FOHPFR, including heating power, filling ratio, wind speed, and inclination angle. To sum up, the findings of this paper contribute a promising approach to effective thermal management in the presence of high-power density.

## 2. Structural Design and Experimental System

### 2.1. Structure Design of the 3D-Printed FOHPFR

**Figure 1:**
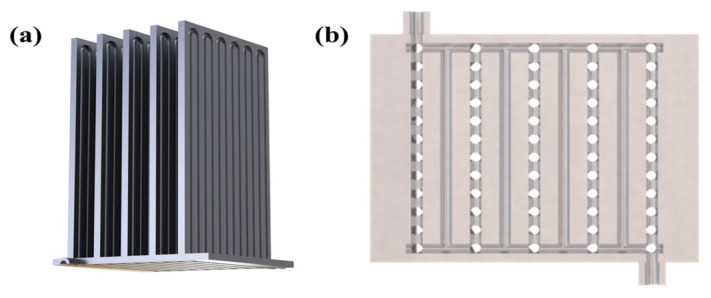
(**a**) Structural diagram and (**b**) bottom structure of the 3D-printed FOHPFR.

**Figure 2:**
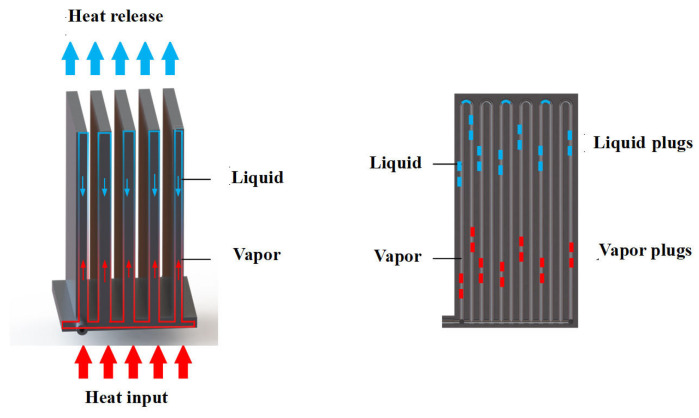
Working principle of the 3D-printed FOHPFR.

Figure 1a presents a schematic diagram of the 3D-printed FOHPFR as proposed in this paper. The rectangular straight fins with equal cross-section are embedded with flat-plate OHPs consisting of 6 elbows. The bottom structure of the FOHPFR is shown in Figure 1b, where the internal channels with circular cross-sections constitute multi-path parallel circuits. Acetone is taken as the experimental working fluid. During the experiment, the evaporation section is located at the bottom of the FOHPFR, while condensation is carried out on the top of the fins. Figure 2 shows the working principle of the FOHPFR. The working fluid sealed within OHP is distributed randomly as a series of vapor–liquid slugs under vacuum because of the significant role of surface tension. The liquid slug starts to evaporate due to heating and causes the local pressure in the evaporation section to increase, which drives the interior working fluid to the condensation section. Meanwhile, the condensation of vapor induces a low pressure in the condensation section, which leads to a significant thermal driving pressure difference between these two sections. In this case, the working fluid oscillates repeatedly, thus achieving the high efficiency of heat transfer.

The structural parameters of the FOHPFR are listed in Table 1.

The diameter of OHP is essential for the oscillation and heat transfer performance. Due to surface tension, the working fluid inside the pipe develops continuously distributed vapor–liquid plugs only when the pipe diameter is small enough. However, an excessively small diameter increases the wall resistance and disrupts the operation of the OHP. According to Hosoda et al. [31], the diameter of OHP usually conforms to the following inequality:

(1) $$0.7\sqrt{\frac{\sigma }{g\left(\rho_{l}-\rho_{v}\right)}}\leq D\leq 1.8\sqrt{\frac{\sigma }{g\left(\rho_{l}-\rho_{v}\right)}}$$

where *D* represents the inner diameter, σ indicates the surface tension of the working fluid, $\rho_{l}\;\mathrm{and}\;\rho_{v}$ are referred to as the density of the liquid phase and vapor phase of the working fluid, respectively, and g means the gravitational acceleration. Using the above analysis, combined with the relevant research results, the diameter of the OHP in this design is determined to be 2 mm, which meets the size requirements of the OHP [32].

Based on the experimental experience in 3D printing [30], the wall thickness of the 3D-printed flat-plate OHP is supposed to be no less than 1.5 mm generally. Thus, the fin thickness is set to 5 mm. The fin spacing is formulated as follows:

(2) $$b=1.5\sqrt[{4}]{\frac{L\upsilon^{2}}{\beta \Delta tPr}}$$

where *b* represents the spacing of fins, *L* indicates the width of the fin, *ν* denotes kinematic viscosity, *β* is referred to as the coefficient of fluid expansion, and Δ*t* indicates the difference between the wall temperature of the fin and the ambient temperature. The spacing between the straight fins of equal cross-section is determined to be 5.7 mm in accordance with the above equation. The relationship between heat dissipation and fin height is expressed as follows:

(3) $$\Phi =\lambda A_{c}m\left(t_{0}-t_{f}\right)th\left(mH\right)$$

In which,

(4) $$m=\sqrt{\frac{2h\left(L+\delta \right)}{\lambda L\delta }}$$

where *h* represents the convective heat transfer coefficient, *L* indicates the fin width, λ denotes the thermal conductivity, and δ refers to the fin thickness.

In general, the fin efficiency used in engineering works is 0.8 at minimum. Therefore, the fin height is calculated as *H* ≈ 76 mm. It is worth noting that as the length of the OHP increases, there is a rise in the distance at which the vapor slug flows toward the condensing section, and the heat flux increases accordingly. It is inferred that increasing the length of OHP is effective in improving the heat transfer performance to some extent [33]. Therefore, to ensure excellent oscillation and heat transfer performance, an appropriate adjustment is made on the basis of this equation, and the fin height is determined as 90 mm.

**Figure 3:**
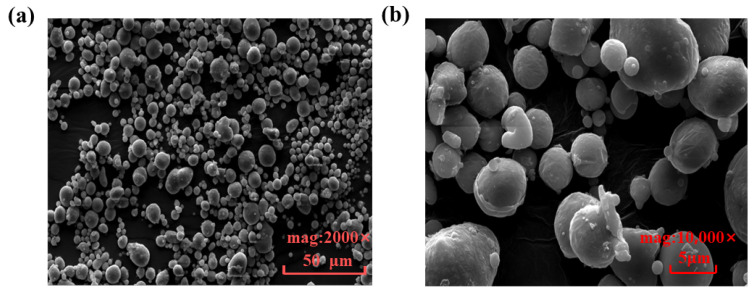
SEM image of AlSi_10_Mg metal powder. (**a**) Magnified by 2000 times, (**b**) Magnified by 10,000 times.

Notably, the component material is the medium for external heat input, which necessitates a high performance in heat conductivity and requires that no reaction involves the internal substance. Meanwhile, the requirements on rigidity and mechanical strength must be satisfied under the experimental conditions as described in this paper. According to the specifications of material selection for SLM, powdered AlSi_10_Mg is taken as the material used for 3D printing in this study. Scanning electron microscopy (SEM) was performed on AlSi_10_Mg metal powder, and Figure 3 shows the experimental results. The spherical powders are uniform in particle shape, which ensures excellent flowability. This makes it possible to spread the powders into a thin layer during the printing process, thereby improving the dimensional accuracy and surface quality of the 3D-printed device. Also, spherical powders are of high density and capable of resisting agglomeration, which ensures the high precision and stability of 3D printing. More importantly, the low-density aluminum alloy radiator performs well in heat conductivity and dissipation, which aligns with the future trend of development for portable, lightweight electronics.

### 2.2. Manufacturing of the 3D-Printed FOHPFR

**Figure 4:**
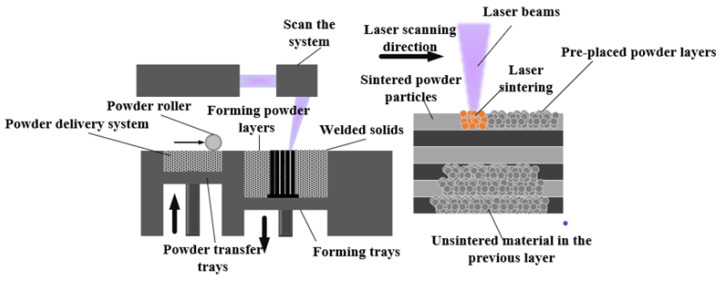
Manufacturing flowchart of the 3D-printed FOHPFR.

In this study, the 3D-printed technology SLM is applied for the fabrication of FOHPFR. This technology uses the fiber laser with excellent beam patterns, which features extremely high laser power and the capability to completely melt the metal powder [34]. Figure 4 illustrates the manufacturing process and principle of the 3D-printed FOHPFR. SolidWorks is first applied to construct the required solid device model, and the 3D model is divided into multiple 2D cross-sections to determine the scanning path. Scraper is used to spread the AlSi_10_Mg powder with a diameter of about 20 µm evenly across the laser processing area, and the metal powder is selectively melted by the laser beam controlled by the computer. When the molten metal powder solidifies, the solid body with a corresponding cross-section is obtained. After that, the lifting platform is lowered by a unit thickness, and the above operation is repeated. Finally, the same 3D entity as the designed model is stacked layer by layer. During this process, Argon gas is cut off as the protective gas to avoid the oxidation of sintered aluminum particles.

### 2.3. Experimental System and Measurement

Due to the layer-by-layer printing as a characteristic of SLM technology, there is residue metal powder that is not sintered by the laser inside the FOHPFR. Therefore, it is necessary to dry clean the powder remaining in the channels of the device before testing. Firstly, the air pump outlet is connected to the fluid-filling port of the FOHPFR, and the pressure inside the pump is adjusted to expel the residual metal powder through a powder discharge port. Then, the FOHPFR is placed in an ultrasonic cleaner, with deionized water and acetone solution added in sequence for 15 min each to remove the residual powder and surface contaminants from the FOHPFR. This process is repeated several times. After the cleaning process is complete, a blow-drying oven is used to dry the FOHPFR, preventing the working fluid from contamination by the residual cleaning liquids to avoid any compromise on the heat transfer performance of the heat pipe. Finally, an aluminum-based repair compound is prepared as instructed to seal the fluid-filling and powder discharge ports of the FOHPFR.

**Figure 5:**
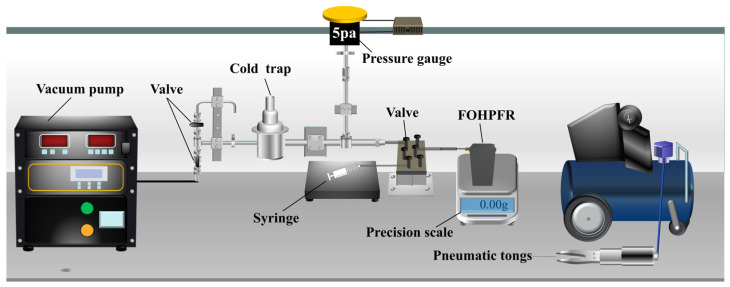
Schematic diagram of the liquid filling system.

Liquid filling represents a crucial part of the experiment performed on OHP. Figure 5 illustrates the filling system used for this experiment. Considering the particularity of the working environment inside the OHP, it is vacuumed before the working fluid is filled. As shown in this figure, the vacuum pump is used to vacuum the circuit and pipe. The cooled trap (cooling system) is a protective device for the molecular pump, which can be used to condense the input gas or liquid rapidly, thus preventing the molecular pump from internal damage. The pressure inside the circuit is monitored in real time by the pressure gauge and is used to determine whether the vacuum degree within the heat pipe is sufficient for the filling of working fluid (less than 5 Pa) [30,35].

**Figure 6:**
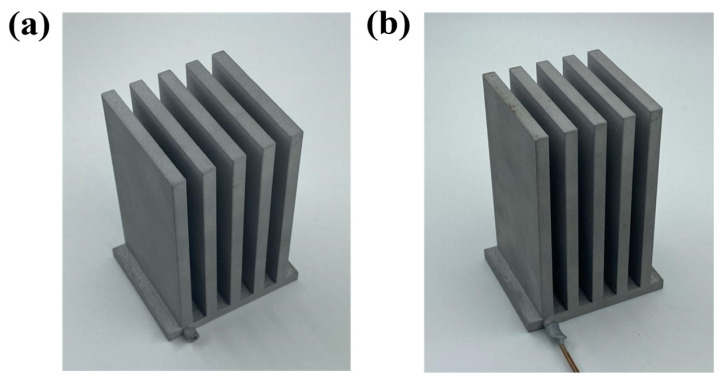
(**a**) Before sealing and (**b**) after sealing of the 3D-printed FOHPFR.

In this experiment, the performance of the 3D-printed FOHPFR is tested at 5 different filling ratios (30%, 40%, 50%, 60%, and 70%). The filling ratio is defined as the ratio between the volume of loaded working fluid (i.e., acetone in this case) and the inner space of the device [36,37]. The syringe with working fluid inside is connected to the device via a valve. A precision scale is applied to indicate the mass of the fluid entering the FOHPFR, which facilitates the precise control of filling. During this process, the scale is first zeroed, and the valve is closed. As the valve connecting the syringe is opened up, the working fluid within the syringe is driven into the device by the atmospheric pressure. When the reading of the scale is found constant, the indication is the total mass of the working fluid when the FOHPFR is fully filled. Accordingly, the filling ratio is obtained as a ratio of the mass of the working medium filled to this value. When the liquid filling is completed, pneumatic tongs are used to cut the pipe, and the inlet and outlet of the FOHPFR are sealed using an aluminum-based repair agent. Figure 6 presents the before and after sealing of the 3D-printed FOHPFR.

**Figure 7:**
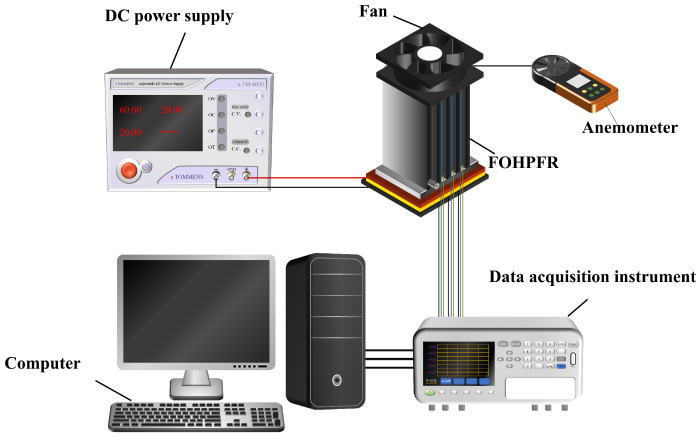
Schematic diagram of the experimental system.

Figure 7 illustrates the experimental test system in detail. During the experiment, a grooved copper block was tightly pressed in place between the heating plate and the FOHPFR. The groove is purposed to facilitate the measurement of temperature at the heat source. Thermal conductive silicone grease was evenly applied to each contact surface, which reduces the thermal resistance upon contact. A DC power supply (HCP1024 from Shenzhen Henghui, China) was used to heat the heating plate located at the bottom of the FOHPFR. The maximum heating power was 850 W, and the device was used to align the voltage (0–100 V) and current (0–8 A) with the requirement using a port. The heating plate was wrapped with insulation cotton to minimize heat loss. Meanwhile, a fan placed at the top of the fins is used to remove heat via forced convection. The speed of the fan is determined by an anemometer, ranging between 1.5 m/s and 3.5 m/s. A data acquisition instrument is applied to collect temperature-related data from the thermocouples and then transmit it to the computer. Once the data acquisition instrument is switched on, the reading of the thermocouple is checked for any anomalies. When detected, it warrants a prompt replacement.

**Figure 8:**
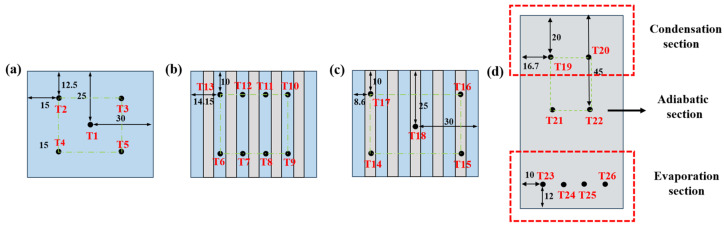
The distribution of thermocouples at the (**a**) bottom, (**b**) evaporation end, (**c**) condensation end and (**d**) fins of the FOHPFR.

To quantitatively evaluate the heat dissipation performance of the 3D-printed FOHPFR, multiple thermocouples are evenly deployed at the bottom, evaporation, and condensation sections of the radiator, as shown in Figure 8. Additionally, 8 thermocouples are placed along the height of straight fins to monitor oscillation within the flat-plate OHP. During the experiment, the heating power ranges from 20 W to 140 W, with the heating step set to 20 W. Each working condition lasts for 10 min to ensure that the radiator reaches a stable operation state. Both thermal resistance and heat source temperature are important indicators to evaluate the heat transfer performance of the finned radiator. They are obtained via the following equations, respectively.

(5) $$T_{s}=\frac{1}{5}\sum_{i=1}^{5}T_{i}$$

(6) $$T_{h}=\frac{1}{8}\sum_{i=6}^{13}T_{i}$$

(7) $$T_{c}=\frac{1}{5}\sum_{i=14}^{17}T_{i}$$

(8) $$R=\frac{T_{h}-T_{c}}{Q}$$

where $T_{s}$ represents the heat source temperature, $T_{h}$ indicates the evaporation terminal temperature, $T_{c}$ denotes the condensation terminal temperature, Q refers to the heating power, and *R* means the thermal resistance.

### 2.4. Analysis of Measurement Errors

The Joule heating generated by the DC power supply is determined using the following formula:

(9) Q=U⋅I

where U represents the voltage, and I denotes the current.

The calculation of the absolute error associated with the heat input to the evaporator is as follows:

(10) $$\frac{\delta Q}{Q}=\sqrt{{\left(\frac{\delta U}{U}\right)}^{2}+{\left(\frac{\delta I}{I}\right)}^{2}}$$

Analysis indicates that the absolute and relative errors for voltage are 0.02% and ±10 mV (±0.01 V), respectively, and for current, they are 0.036% and ±1 mA (±0.001 A). Consequently, these values lead to an absolute and relative error in the heat measurement of Q = 0.04% and 0.056 W, respectively.

As previously noted, the FOHPFR was equipped with 26 K-type thermocouples, each with a relative error of T = ±0.1 °C.

## 3. Results and Discussion

### 3.1. Surface Wettability and SEM Characterization of the 3D-Printed FOHPFE

**Figure 9:**
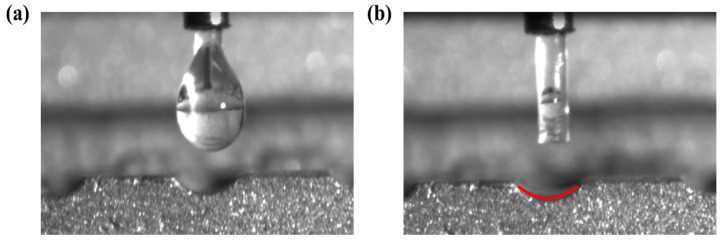
Comparison of acetone droplet before and after contact with the channel surface. (**a**) before, (**b**) after.

Surface wettability plays a significant role in the flow and heat transfer performance of the heat pipe finned radiator. The main reason is that the micro-nano structure of superhydrophilicity enhances the evaporation rate of the thin liquid film in the evaporation section of OHP [25]. To explore the surface wettability of the AlSi_10_Mg laser sintered surface, a contact angle measuring instrument is used to test the wetting performance of the grooves in the FOHPFR, and the results are shown in Figure 9. When the acetone drop falls onto the inner surface of the OHP, it diffuses rapidly in less than 1 s due to capillary force, thus forming an extremely thin liquid film with a contact angle of almost 0°. This indicates an excellent wetting performance of the OHP surface. Therefore, the 3D-printed AlSi_10_Mg microchannel has excellent surface wettability to achieve efficient heat transfer.

**Figure 10:**
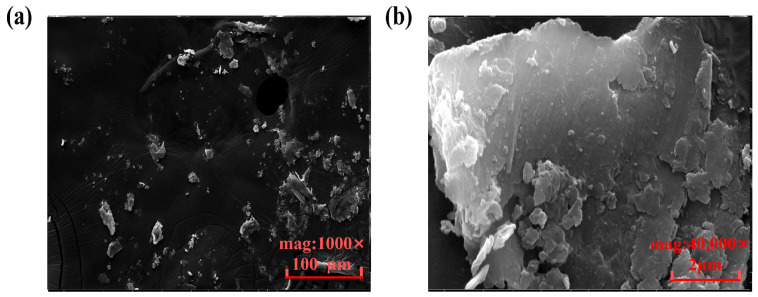
SEM characterization of the interior of the 3D-printed FOHPFR. (**a**) Magnified by 2000 times, (**b**) magnified by 40,000 times.

In addition, SEM is performed to observe and analyze the surface structure of the AlSi_10_Mg laser sintering. The result is exhibited in Figure 10. It can be seen that the 3D-printed FOHPFR has a rough inner surface. This is because the metal powder sticks together when laser sintered, which gives rise to many micropores and bulges on the surface, thus making it appear rough [38]. And the rough inner surface can effectively increase the contact area between acetone and the printing surface. This provides a strong capillary driving effect for the working fluid and is conducive to strengthening heat transfer [39].

### 3.2. Performance Improvement Verification of Finned Radiator

**Figure 11:**
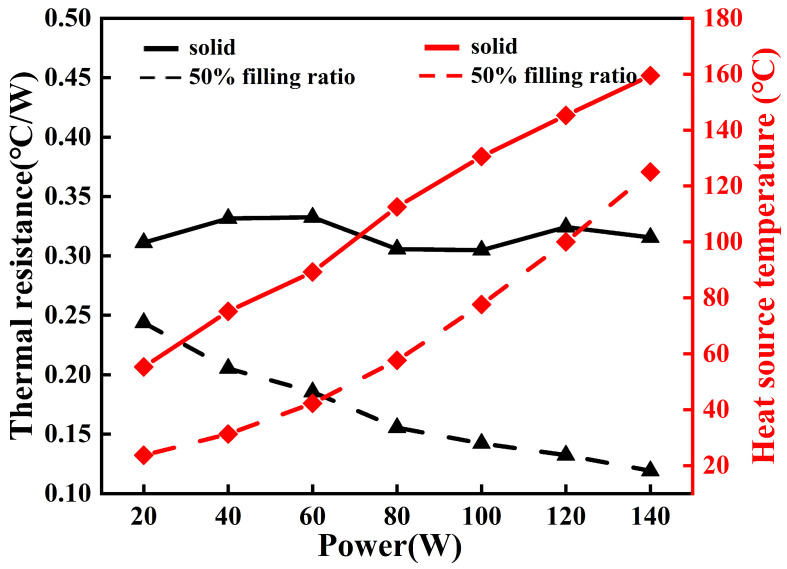
Performance comparison of the 3D-printed FOHPFR and solid finned radiator.

To evaluate the heat dissipation performance of the 3D-printed FOHPFR, its performance parameters are compared against the solid finned radiator with the same structure and same material, and the results are shown in Figure 11. In this figure, the solid and dashed lines represent the performance parameters of the solid finned radiator and the FOHPFR at the liquid filling ratio of 50%, respectively, and the black and red lines indicate the heat resistance and heat source temperature of the radiator, respectively. It can be seen that the heat resistance of the solid finned radiator remains constant in the range of 0.3–0.35 °C/W when the heating power varies from 20 W to 140 W. However, the heat resistance of the FOHPFR shows a trend of continuous decrease with heating power, reaching a minimum of 0.11 °C/W at 140 W. In addition, the temperature at the heat source of the solid finned radiator is consistently higher compared to the 3D-printed FOHPFR, and the difference can reach 35 °C given the maximum heating power. Therefore, the FOHPFR demonstrates a 65.6% reduction in thermal resistance and a 21.8% decrease in heat source temperature at 140 W heating power.

The main reason for this phenomenon is that the fins of POHPFR are embedded with a flat-plate OHP structure, and OHP uses the working fluid to reciprocate and circulate in the pipe to achieve highly efficient heat transfer. Furthermore, with the increase in heating power, the heat absorption of the working fluid in the OHP increases, and the phase transformation speed of the vapor–liquid plug is accelerated. As a result, the reciprocating oscillation and circulation of the working fluid are more intense, and the heat transfer resistance is further reduced. Therefore, the 3D-printed FOHPFR, as proposed in the paper significantly outperforms the solid finned radiator in heat dissipation performance, which provides a lighter and more efficient heat dissipation solution for high-power components.

### 3.3. Response Performance of the 3D-Printed FOHPFR

**Figure 12:**
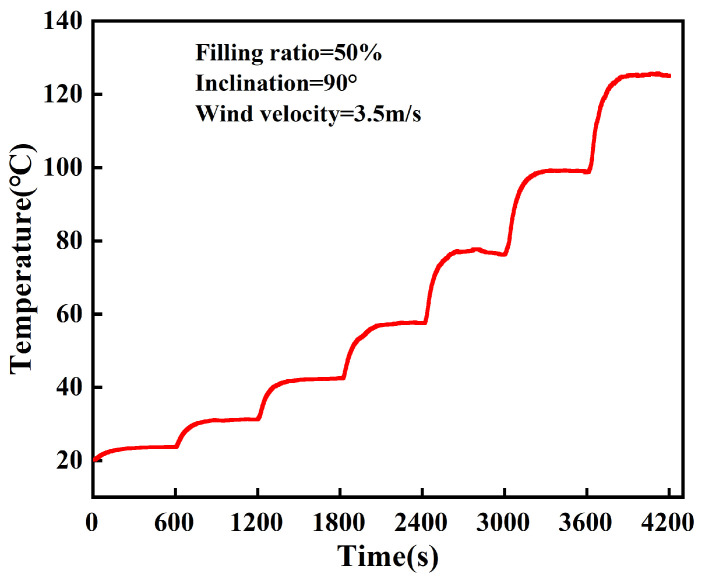
Heat source temperature variation in FOHPFR at a filling rate of 50%.

In order to study the instantaneous heat absorption capacity of the 3D-printed FOHPFR, a response performance test is conducted on the FOHPFR with the heating power ranging from 20 to 140 W. Figure 12 shows the heat source temperature variation in FOHPFR. Here, the liquid filling ratio is set to 50%, and the wind speed at the condensation section is 3.5 m/s. Once powered, the FOHPFR absorbs a large amount of heat quickly, and it enters a steady state in about 170 s. As the heating power increases, the heat source temperature of FOHPFR shows gradient-like changes. This indicates that the 3D-printed FOHPFR is thermally responsive and consistent in heat transfer performance.

**Figure 13:**
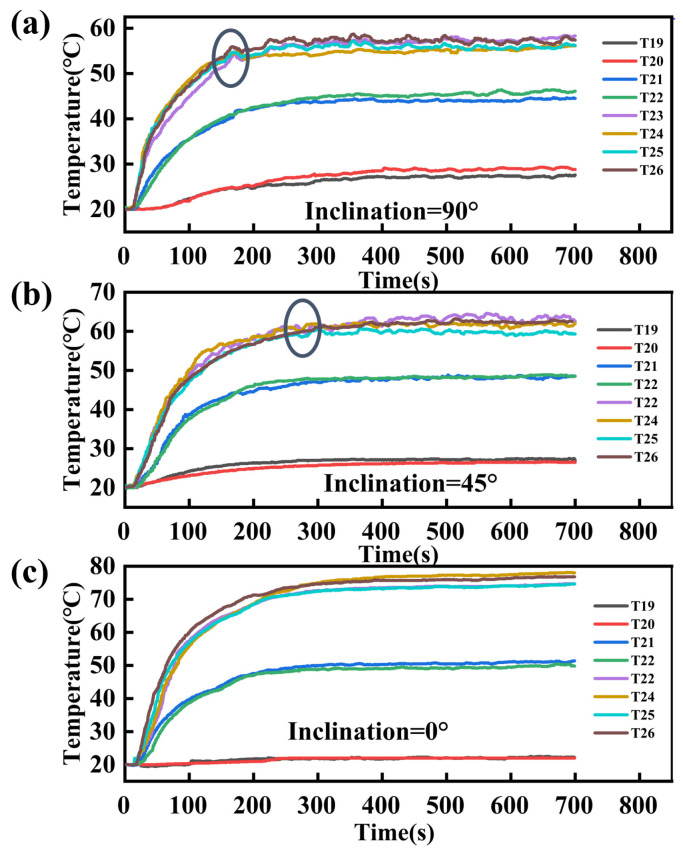
Oscillating curve of flat-plate OHP at different inclination angles. (**a**) 90°, (**b**) 45° and (**c**) 0°.

Figure 13 presents the starting performance of the flat-plate OHP given different inclination angles. Figure 13a shows the temperature oscillation curve when the inclination angle is 90°, and heating power is 140 W. Obviously, temperature rise occurs at the evaporation, condensation, and adiabatic sections when the flat-plate OHP has not started yet, which is attributed to the heat conductivity of metallic materials. When the temperature drops abruptly at the evaporation section, as circled in the figure, the flat-plate OHP starts to operate. This is because when heat is transferred to the OHP, the working fluid inside the channel at the evaporation section starts to evaporate, but the pressure generated at this time is insufficient to drive the working fluid to the condensation section. At about 170 s, there is sufficient pressure accumulated at the evaporation section, and the working fluid undergoes a significant circulation abruptly. Consequently, vapors are replaced with the low-temperature working fluid at the evaporation section, which leads to a sharp decline in temperature at this section. After that, the flat-plate OHP reaches an equilibrium between the input and output of heat due to cooling, heat transfer, metallic heat conduction, and vapor–liquid interface oscillation. Therefore, the temperature shows long-term stability despite some minor fluctuations. At this time, the pattern of heat transfer shifts from metallic thermal conduction to phase change in the working fluid, which significantly improves the heat dissipation performance.

However, as the inclination angle of the FOHPFR decreases, the oscillation of the working fluid and heat transfer performance of OHP decrease progressively. This is because a decline in the inclination angle mitigates the effect of gravity and reduces the rate at which the working fluid flows back. As shown in Figure 13b, it takes about 300 s for the FOHPFR to work stably when the inclination angle is 45°. Furthermore, it is difficult for the working fluid to start to oscillate when the FOHPFR is placed horizontally. Therefore, no significant fluctuation is observed from the temperature curve in Figure 13c.

When comparing the startup curve of the OHP to the response curve of the FOHPFR, it is noted that the heat reaches the OHP through the radiator’s substrate after about 20 s of heating, and the OHP exhibits a temperature rise. Approximately 170 s into the process, the OHP is fully activated at about 170 s, at which point the POHPFR completes its response and enters a stable operating state. Therefore, the startup characteristics of the OHP are crucial in determining the response performance of the FOHPFR.

### 3.4. Heat Transfer Performance Analysis of the 3D-Printed FOHPFR

Different from the traditional solid finned radiators that rely on heat conduction and convective heat transfer, the performance of the 3D-printed FOHPFR developed in the paper is largely determined by the cyclic oscillation of the working fluid inside the flat-plate OHP. Therefore, the main parameters affecting the heat transfer performance of the OHP, including the liquid filling ratio, inclination angle, and the operating conditions at the cold terminal, are crucial for the performance of FOHPFR.

**Figure 14:**
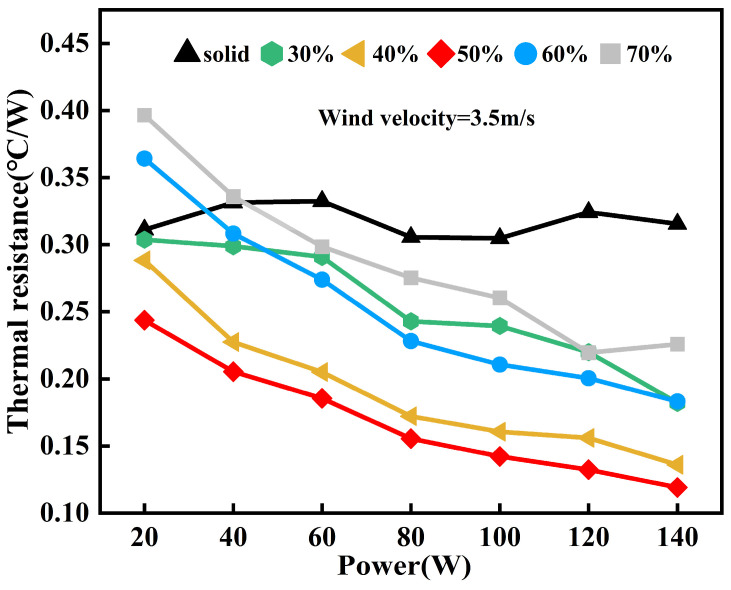
Impact of filling ratio on the heat transfer performance of the 3D-printed FOHPFR.

The impact of the filling ratio on the heat transfer performance of the 3D-printed FOHPFR is explored first, and the results are shown in Figure 14. In this figure, the filling ratio is set to 30%, 40%, 50%, 60%, and 70%, respectively, and the wind speed at the cold terminal is set to 3.5 m/s. On the one hand, the thermal resistance of the FOHPFR diminishes gradually, regardless of heating power, when the liquid filling ratio rises from 30% to 50%. However, as the filling ratio is further increased, the deterioration in heat transfer performance begins. This is because the low level of liquid filling restricts the evaporative phase change and heat transfer of the working fluid in the FOHPFR, which hinders the heat transfer effect. However, a strong flow resistance is formed in the FOHPFR when the liquid filling ratio is excessively high, which inhibits the gas working fluid from reaching the condensation section for heat release, thus reducing the heat dissipation capacity of the flat-plate OHP. Therefore, there is an optimal filling ratio of 50%, under which the reflux of the working fluid becomes uniform, sufficient, and smooth, and the balance between potential heat and heat dissipation reaches the most satisfactory level so that the optimal working performance of the 3D-printed FOHPFR is achieved. On the other hand, when the heating power exceeds 40 W, the thermal resistance of the 3D-printed FOHPFR is always lower compared to the solid finned radiator, indicating the successful startup of the OHP. In addition, with the increase in heating power, the thermal resistance diminishes gradually. It is worth noting that given a heating power of 140 W and a liquid filling rate of 50%, the minimum thermal resistance of the 3D-printed FOHPFR reaches 0.11 °C/W, which is about 65.6% lower than that of the solid finned radiators under the same condition of heating.

**Figure 15:**
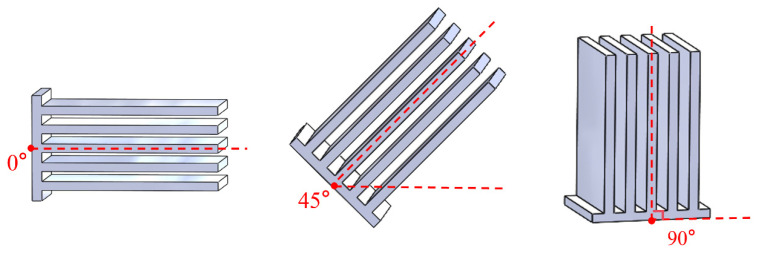
Schematic diagram of the inclination angle.

**Figure 16:**
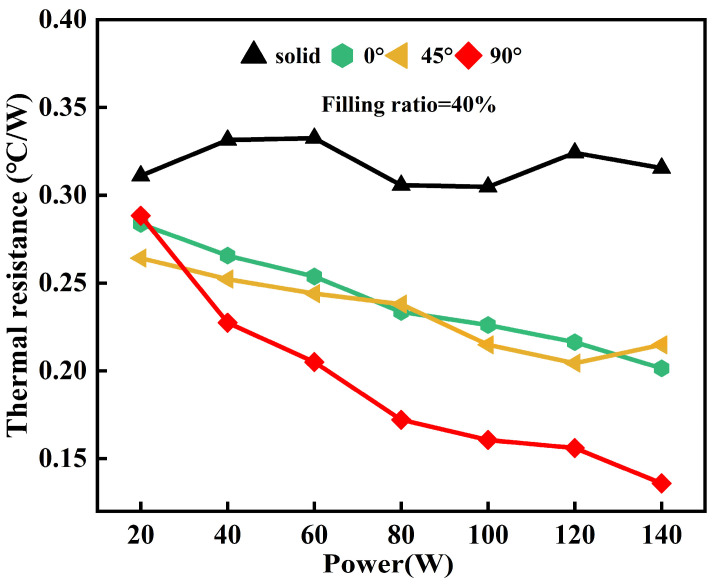
Impact of inclination angles on the heat transfer performance of the 3D-printed FOHPFR.

When the OHP is at different inclination angles, there is a variation in the driving force or resistance to the circulation of working fluid inside the pipe, which further affects the heat transfer performance of the device. The inclination angle is defined as the intersection angle between the central axis of the FOHPFR and the horizontal plane, as shown in Figure 15. In practice, the radiator may be placed at different working angles. Therefore, one of the focuses of research on heat pipe finned radiators is to make them adapt to different working angles. Figure 16 shows the heat transfer performance of the 3D-printed FOHPFR at different inclination angles, given a filling ratio of 40% and a wind speed of 3.5 m/s at the cold terminal. As can be seen, the heat resistance of the 3D-printed FOHPFR is effectively reduced compared to that of the solid finned radiator at all inclination angles. This indicates that the flat plate OHP in the radiator has started successfully, and the FOHPFR possessed good adaptability to various working angles. In addition, when the inclination angle reaches 0°or 45°, the heat dissipation performance of the FOHPFR is clearly inferior to that at 90°. This is due to the fact that at a large inclination angle, the cycles of vaporization and condensation of the working fluid can be completed more quickly under the action of gravity. Meanwhile, with the increase in power, the heat dissipation capacity is significantly improved at the inclination angle of 90°.

**Figure 17:**
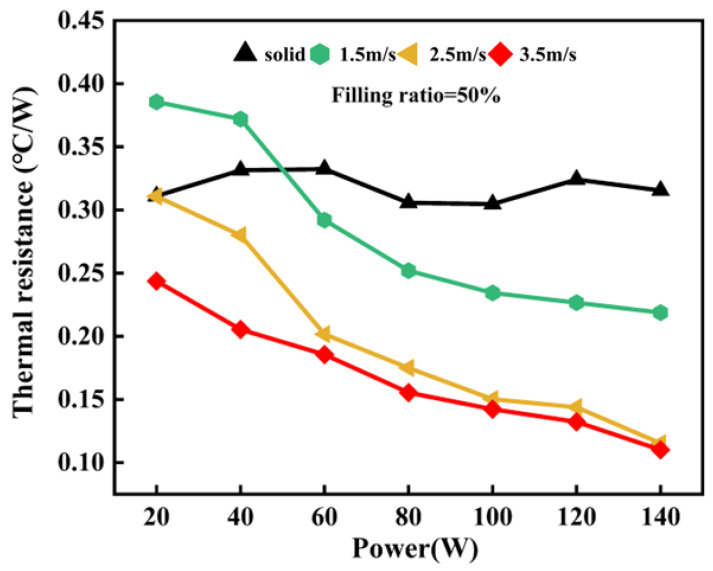
Impact of wind speed on the heat transfer performance of the 3D-printed FOHPFR.

Figure 17 shows the changes in thermal resistance of the finned radiators given different wind speeds at the cold terminal. To be specific, the wind speed is set to 1.5 m/s, 2.5 m/s, and 3.5 m/s, respectively, and the filling ratio is 50%. When the wind speed at the cold terminal is raised from 1.5 m/s to 2.5 m/s, there is a significant improvement in the convective heat transfer and condensation effect of the working fluid at the top of the fins. Therefore, thermal resistance is effectively reduced for the 3D-printed FOHPFR. Specifically, the thermal resistance of the FOHPFR declines from 0.23 °C/W to 0.12 °C/W at a heating power of 140 W. However, with a further increase in wind speed at the cold terminal, the pressure difference caused by evaporation and condensation in the OHP is gradually maximized, and the influence of wind speed becomes negligible.

## 4. Conclusions

In this paper, a novel, efficient finned radiator based on arrayed flat-plate OHPs is proposed, and 3D-printed technology is used to realize the customized and lightweight fabrication of the aluminum finned heat exchanger, which provides a solution for efficient thermal management of high-power devices. By comparing its working performance with the solid finned radiator, the superior heat transfer performance of the 3D-printed FOHPFR is demonstrated. On this basis, a further study is conducted on the impact of liquid filling ratio, inclination angle, and working condition at the condensation section, which leads to the following main conclusions:

1. Compared with the solid finned radiator with the same structural parameters, there is a significant improvement in the thermal performance of the 3D-printed FOHPFR. Given a heating power of 140 W, the minimum heat resistance and the heat source temperature of the 3D-printed FOHPFR are 65.6% and 21.8% lower than those of the solid finned radiator, respectively.
2. The starting performance of the flat-plate OHP is essential for the responsiveness of the 3D-printed FOHPFR. With the increase in heating power, the heat source temperature curve of the device shows gradient-like changes, indicating that the 3D-printed FOHPFR performs well in instantaneous heat absorption and steady-state heat transfer.
3. An optimal filling ratio of 50% was identified for the vertical 3D-printed FOHPFR, with minimal thermal resistance achieving 0.11 °C/W. Moreover, the 3D-printed FOHPFR demonstrates good adaptability to various operating angles. However, the heat dissipation performance deteriorates to a certain extent when the inclination angle is set to 0°.
4. Due to the internal microchannels along with their rough sintered internal surfaces in the 3D-printed FOHPFR, an extensive wetting area and capillary force are provided for the oscillation working fluid, which effectively enhances the heat dissipation capacity of the device.

## Figures and Tables

**Table 1:** Structural parameters of the 3D-printed FOHPFR.

Name	Size (mm)
Diameter of OHP	2
Fin width	50
Fin height	90
Fin spacing	5.7
Base thickness	5
Base area	50 × 60

## Data Availability

The data presented in this study are available upon request from the corresponding author.

## Nomenclature

3D	three-dimensional
OHP	oscillating heat pipe
FOHPFR	flat-plate oscillating heat pipe finned radiator
SLM	selective laser melting
DC	direct current
SEM	scanning electron microscopy
